# Development of partial abiotic stress tolerant *Citrus reticulata* Blanco and *Citrus sinensis* (L.) Osbeck through *Agrobacterium*-mediated transformation method

**DOI:** 10.1186/s43141-019-0014-3

**Published:** 2019-12-16

**Authors:** Nazmul Hasan, Mohammad Kamruzzaman, Shariful Islam, Hammadul Hoque, Fahmid Hossain Bhuiyan, Shamsul H. Prodhan

**Affiliations:** 1 Department of Genetic Engineering and Biotechnology, Shahjalal University of Science and Technology, Sylhet, 3114 Bangladesh; 2 Department of Molecular Biology and Genetic Engineering, Sylhet Agricultural University, Sylhet, 3100 Bangladesh

**Keywords:** *Citrus*, *Agrobacterium*, Transformation, Regeneration, PCR, Abiotic stress

## Abstract

**Background:**

Recent studies indicate that farmers are facing several challenges due to biotic and abiotic stresses like diseases, drought, cold, and soil salinity which are causing declined *Citrus* production. Thus, it is essential to improve these varieties which would be resistant against biotic and abiotic stresses as well as high yielding. The transformation of abiotic stress tolerant genes in *Citrus* species is essential for using areas affected by abiotic stresses. This study was aimed to improve resistance of *Citrus reticulata* Blanco and *Citrus sinensis* (L.) Osbeck to abiotic stresses by transferring *PsCBL* and *PsCIPK* genes through *Agrobacterium*-mediated transformation.

**Results:**

Abiotic stress tolerant *PsCBL* and *PsCIPK* genes isolated from *Pisum sativum* were transformed into two *Citrus* species, *Citrus reticulata* Blanco and *Citrus sinensis* (L.) Osbeck, through *Agrobacterium*-mediated transformation method. Mature seed-derived calli of two Species were infected with *Agrobacterium tumefaciens* LBA4404 harboring *PsCBL* and *PsCIPK* genes. The infected calli were co-cultured in dark condition and later on washed with antibiotic solution and transferred to selection medium. Preliminary resistant calli were recovered and regenerated to plantlets. Maximum regeneration rate was 61.11 ± 1.35% and 55.55 ± 1.03%, respectively. The genetic transformation was confirmed by performing *β* glucuronidase (GUS) assays and subsequent PCR amplification of the GUS gene. The transformation rates of the two cultivated species were higher than previous reports. Maximum transformation frequencies were found when bacterial OD_600_ was 0.5 and concentration of acetosyringone was 150 μM. In-vitro evaluation of drought and salt tolerance of transgenic plantlets were done, and transgenic plantlets showed better performance than the control plants.

**Conclusions:**

The present study demonstrates that transformation of *Citrus* plants with *PsCBL* and *PsCIPK* genes result in improved abiotic stress tolerance.

## Background

The genus *Citrus* includes more than 162 species belonging to the order Geraniales, family Rutaceae and subfamily Aurantoideae. *Citrus reticulata* Blanco and *Citrus sinensis* (L.) Osbeck are two primitive species of *Citrus*. *Citrus sinensis* also known as Sweet-orange or Malta, is the most cultivated *Citrus* in the world which accounts for about 70% of the total production. *Citrus reticulata* Blanco is a species of *Citrus* also known as Mandarin, Tangerine, Unshu orange, Comola etc in the Asian Subcontinent. *Citrus reticulata* Blanco and *Citrus sinensis* (L.) Osbeck, both are grown in tropical and semi-tropical areas around the globe for its sweet, juicy, and nutritious fruits. Orange farming has been extending rapidly in some regions of Bangladesh like Sylhet, Panchagarh, Chattogram, and Thakurgaon. Recent studies indicate that farmers are facing several problems due to biotic and abiotic stresses like diseases, drought, cold, and soil salinity which are causing declined *Citrus* production. Thus, it is essential to improve these varieties which would be resistant against biotic and abiotic stresses and would be high yielding. Traditional breeding methods have been used successfully over the years to improve *Citrus*; however, these methods are limited by slow growth, incompatibility, polyembryony, parthenocarpy etc and traditional breeding takes a long time for the incorporation of desirable traits. In-vitro culture made it easy to improve *Citrus* against different abiotic stresses, diseases, low-yield through exploiting somaclonal variations, somatic cell hybridization [1, 2], transformation of high yielding cultivars [3] and also to conserve important *Citrus* genotypes. In plants, the Ca^2+^ is involved in almost all biological processes. Calcium serves as a ubiquitous secondary messenger and regulates a multitude of physiological and developmental processes, including responses to abiotic stress, pathogen defense, and adjustment of ion homeostasis [4–6]. In response to environmental and developmental stimuli, plant cells react with specific temporal changes in cytosolic calcium (Ca^2+^) concentration. Ca^2+^ can serve simultaneously as a messenger and a regulator in so many different processes, that it raises the fundamental question of how specificity in information processing and output determination can be achieved [7–9]. The families of calcineurin B-like (CBL) proteins represent a unique group of calcium sensors and contribute to the decoding of calcium transients by interacting with and regulating the family of CBL-interacting protein kinases (CIPKs). In higher plants, CBL proteins and CIPKs form a complex signaling network that allows for flexible but specific signal response coupling during environmental adaptation reactions [10]. The CBL-CIPK network helps to maintain proper ion balance when abiotic stresses occur. The CBL and CIPK homolog are present in all green lineages, and phylogenomic analysis suggests their expansion from a single CBL-CIPK pair present in the ancestor of modern plants and algae [10]. Thus, the incorporation of these two key genes into the desired plants would improve the resistance against abiotic stresses. This study was aimed to develop resistance of *Citrus reticulata* Blanco and *Citrus sinensis* (L.) Osbeck to abiotic stresses by transferring *PsCBL* and *PsCIPK* genes through *Agrobacterium*-mediated transformation.

## Methods

### Plant materials and media

Orange species *Citrus reticulata* Blanco and *Citrus sinensis* (L.) Osbeck was collected from Citrus Research Station, Jaintapur, Sylhet, Bangladesh, and identification and confirmation of collected samples were done by J.C. Sarker, Senior Scientific Officer, Citrus Research Station, Jaintapur, Sylhet , Bangladesh. The healthy and good quality mature seeds from these orange cultivars were collected. Dehusked mature seeds were sterilized with 70% ethanol for 5 mins, 0.1% HgCl_2_ and Tween 20 for 2 mins, followed by washing with autoclaved distilled water and then dried on sterilized filter paper. The culture medium used in this study was based on MS [11] basal salts and vitamins (Table 1). All plant samples handled in this experiment were maintained in specific culture room to avoid any direct contact with environment, and all the used chemicals and plant materials were discared following bio-safety guideline provided by institute.

**Table 1:** The composition of different media used in this transformation study

Media	Composition of the media
Callus induction media	MS medium supplemented with different concentrations of 2,4-D (0, 0.5, 1.0, 1.5, 2.0, 2.5, 3.0, 3.5, 4.0, 5.0 mg/l)
MS resuspension media	MS salts and vitamins were supplemented with 68 g/l sucrose, 36 g/l glucose, 3 g/l KCl, 4 g/l MgCl_2_, and dH_2_O. pH was adjusted to 5.2.
Infection medium	Infection medium was prepared by adding bacterial culture in MS resuspension medium in an appropriate ratio to adjust OD_600 _ to 0.5.
Co-cultivation media	MS medium was fortified with 30 g/l sucrose, 10 g/l glucose, 3.0 mg/l 2,4-D and dH_2_O. pH was adjusted to 5.2. After autoclaving the MS medium different concentrations of filter-sterilized acetosyringone (0 μM, 100 μM, 150 μM, and 200 μM) were added.
Selection media	MS medium was supplemented with 30 g/l sucrose and 3.0 mg/l 2,4-D followed by adjustment to the required volume with dH_2_O and pH adjustment to 5.8. After autoclave filter sterilized kanamycin 100 mg/l was added.
Regeneration media	MS medium supplemented with different concentrations of BA and NAA.
Rooting medium	MS medium containing no hormone.

### *Agrobacterium* strains and culture conditions

The transformation studies were carried out with *Agrobacterium tumefaciens* LBA4404 harboring the binary vector pBI121/PsCBL and pBI121/PsCIPK containing abiotic stress tolerant *PsCBL* and *PsCIPK* genes, respectively, *uidA* gene encoding β-glucuronidase (GUS) and *nptII* gene encoding neomycin phosphotransferase II conferring kanamycin resistance [12–14]. The single colonies of both strains were cultured in liquid YEP medium containing 100 mg/l kanamycin and grown in a shaker at 200 rpm in dark at 28 °C for 48 h until the OD_600_ reached between 0.4 and 0.8. The bacterial cells were collected with centrifugation at 8000 rpm for 5 mins and suspended in MS resuspension medium in an appropriate ratio to adjust OD_600_ to 0.5 and then different concentrations of acetosyringone (100, 150, and 200 μM) were added.

### Callus induction

The sterilized seeds were inoculated in test tube on MS medium containing 2,4-D at different concentrations (0, 0.5, 1.0, 1.5, 2.0, 2.5, 3.0, 3.5, 4.0, 5.0 mg/l) and incubated at 25 ± 1 °C in 2000 lx light. Fifty seeds were inoculated per replication, and experiments were repeated three times. After 3 weeks only yellowish white colored globular calli were selected and cut into small pieces and subcultured onto fresh callus induction medium (Table 1) for infection with *Agrobacterium.*

### Infection with *Agrobacterium* and co-culture

Fresh calli were taken from previous experiment and subjected to infection by immersing them in the infection medium containing the LBA4404 strain having pBI121/PsCBL sense vector and LBA4404 strain having pBI121/PsCIPK sense vector for 90 mins with intermittent gentle shaking and dried on sterile filter paper for 30 mins. The infected calli were cultured on co-cultivation medium (Table 1) for 3 days in dark conditions at 25 °C ± 1 °C. The co-cultivation medium was fortified with 0, 100, 150, and 200 μM acetosyringone to determine the optimum concentration of acetosyringone to increase the transformation efficiency.

### Washing and inoculation in selection medium

After the appearance of slight growth of *Agrobacterium* around most of the calli were rinsed 8–10 times in sterile distilled water. Followed by washing with sterile distilled water, the calli were washed with the washing solution containing cefotaxime (500, 600, 700, and 800 mg/l) for 5 mins. The antibiotic treated calli were washed with sterile distilled water and dried on sterile filter paper. Then, the calli were transferred onto the selection medium and incubated for 15 days at 25 ± 1 °C in dark. After that, the calli were again sub-cultured on the selection media (Table 1) for 7 days and incubated in 25 ± 1 °C in dark.

### Regeneration of putative transformants and In-vitro evaluation

Fresh and growing calli from the second selection medium were transferred to regeneration medium containing different concentrations and combinations of BA and NAA and were incubated at 25 ± 1 °C in white fluorescent light under 16-h photoperiods and sub-cultured on the same media after 14 days. Shoot proliferation started after 3 weeks and the regenerated shoots were shifted to the rooting media and were maintained at 25 ± 1 °C in white fluorescent light under 16-h photoperiods. In-vitro evaluation of kanamycin resistant-regenerated plantlets was done in MS medium supplemented with different concentrations of NaCl (50, 100, 150, and 200 mM) and 3 mg/l PEG. Tolerant plants were maintained under controlled conditions by sub-culturing at an interval of 21 days regularly.

### GUS histochemical assay of transformed callus

GUS activity was detected as described by Jefferson et al. (1987). Randomly kanamycin-resistant calli of two *Citrus* species were taken from selection medium and washed with autoclaved distilled water to remove adjacent media. The calli were incubated in X-glucuronide (5-bromo-4-chloro-indoyl β-D glucuronide) staining solution at 37 °C for 24 h in dark. The X-glucuronide was broken down by the activity of β-glucuronidase (GUS) gene, which was transferred to the cells of calli by *Agrobacterium tumefaciens*. The stained tissues were rinsed several times with 75% ethanol. Calli stained with indigogenic dye were scored, and stable GUS expression was tested in kanamycin-resistant calli. The transformation efficiency was calculated by percent of GUS-positive calli.

### PCR analysis of transformed regenerated plantlets

The genomic DNA of kanamycin-resistant regenerated plantlets (putative) and control plants of two *Citrus* species were extracted from the leaves by using the modified CTAB method [15]. The 600 bp fragments of the GUS gene were amplified using the following set of primer: Forward 5´TTTGCAAGTGGTGAATCCCGACCT-3´ and Reverse 5´AGTTTACGCGTTGCTTCCGCC AGT-3´ [16]. The PCR reaction was carried out in 25-μl mixture containing 2.5 μl 10X Taq buffer, 1.5 μl 25 mM MgCl_2_ , 1.0 μl dNTPs mix, 6.25 μl 2 μM Forward primer, 6.25 μl 2 μM Reverse primer, 0.2 μl Taq DNA polymerase (5U/μl), 2.3 μl Sterile ddH_2_O and 5.0 μl Template DNA. The PCR reaction conditions were initial denaturation at 95 °C for 5 mins, 35 cycles of denaturation at 95 °C for 1 min, annealing at 54 °C for 1 min, extension at 72 °C for 2 mins, and the final extension at 72 °C for 10 mins. The PCR products were confirmed by running in 1.4% agarose gel ectrophoresis in 1X TBE buffer. 2.0 μl loading dye was mixed with PCR products and loaded in the wells. A marker DNA (1 Kb Sharp DNA Ladder Marker, RBC Bioscience Corporation) was also loaded on one side of the gel and electrophoresis was conducted at 75 V for 50 mins. After completion of electrophoresis, the gel was stained in ethidium bromide for 2 h and placed on UV transilluminator in Gel Documentation System. Finally, the photograph was captured (Nikon D5300).

### Statistical analysis

All the experimental data were collected at regular intervals for analysis and reckoned under statistical basis. Arithmetic mean (A.M.) and standard deviation (S.D.) were calculated by analyzing the data with Microsoft Excel 2007. Standard error (S.E.) was calculated by dividing standard deviation by square root of the total three replications for a single variety.

## Results

**Fig. 1:**
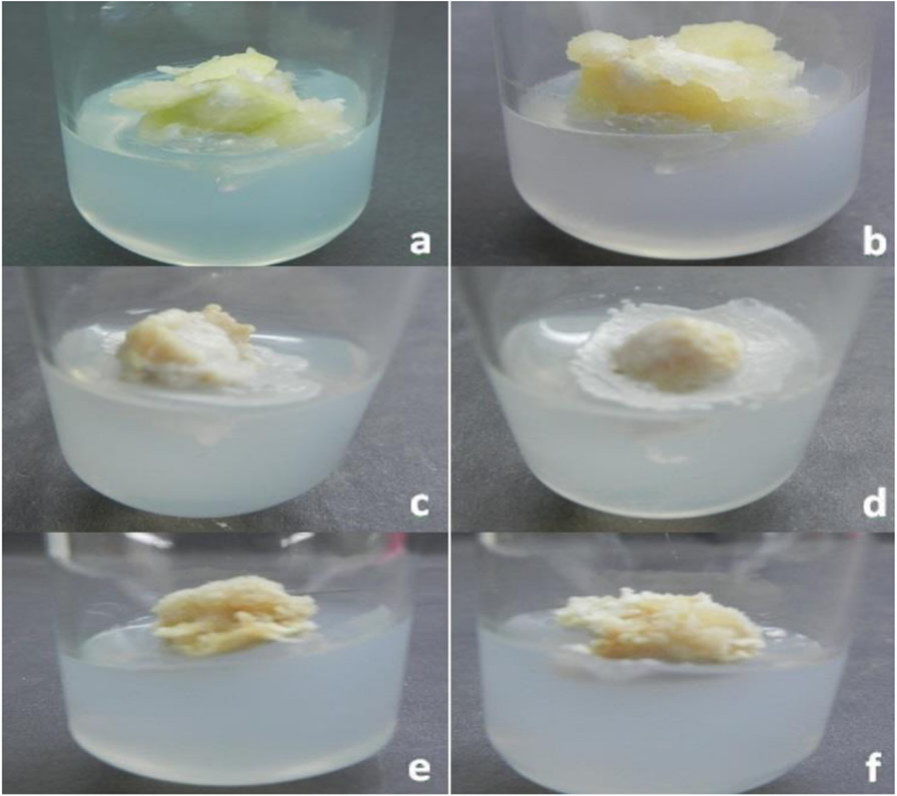
Calli infected with *Agrobacterium* and washed with antibiotic. **a** calli induced from *Citrus reticulata* Blanco; **b** calli induced from *Citrus sinensis* (L.) Osbeck; **c** infected calli of *Citrus reticulata* Blanco; **d** infected calli of *Citrus sinensis* (L.) Osbeck; **e**: antibiotic-treated calli of *Citrus reticulata* Blanco, and **f** antibiotic-treated calli of *Citrus sinensis* (L.) Osbeck

**Fig. 2:**
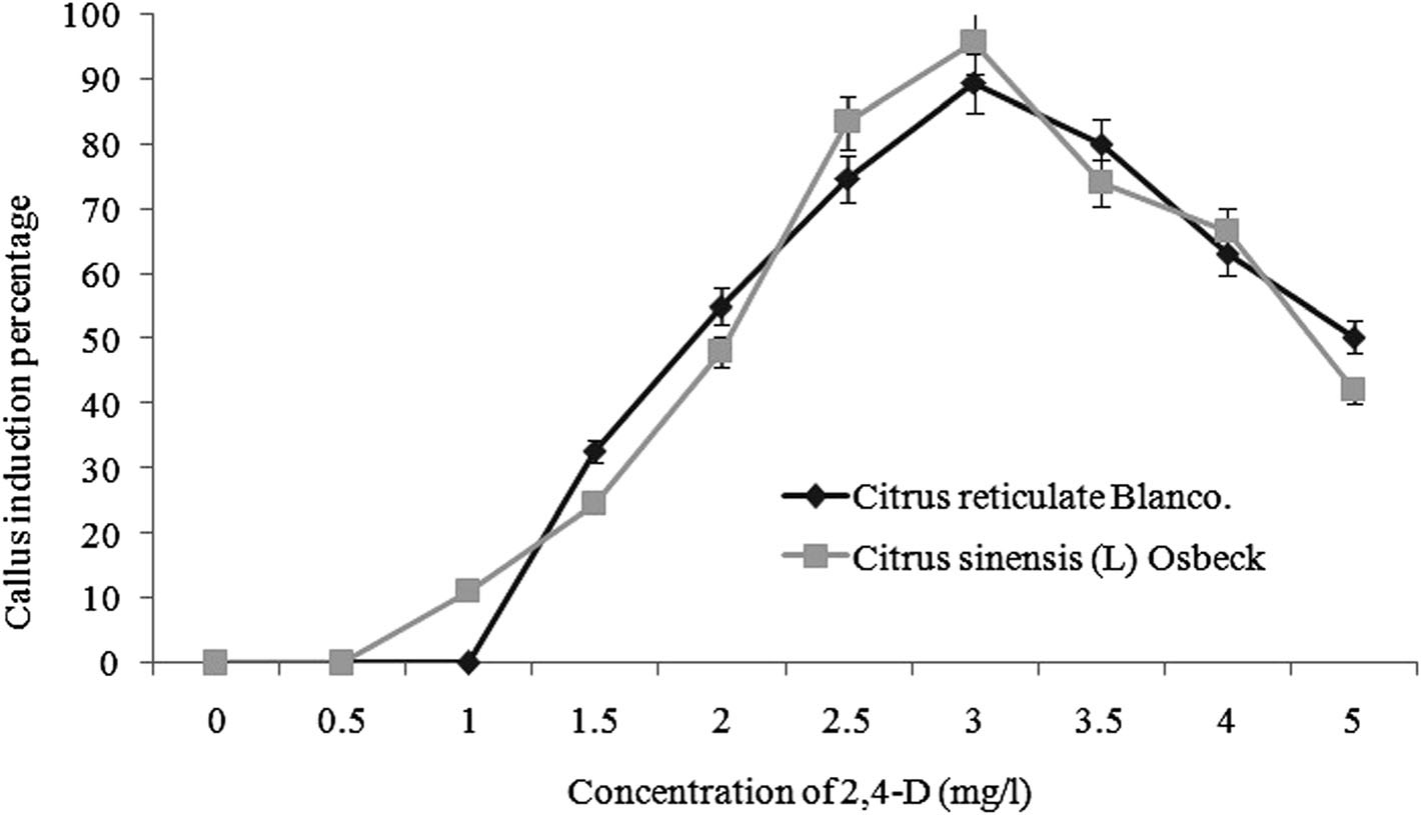
Role of 2,4-D on callus induction in *Citrus reticulata* Blanco and *Citrus sinensis* (L.) Osbeck

**Fig. 3:**
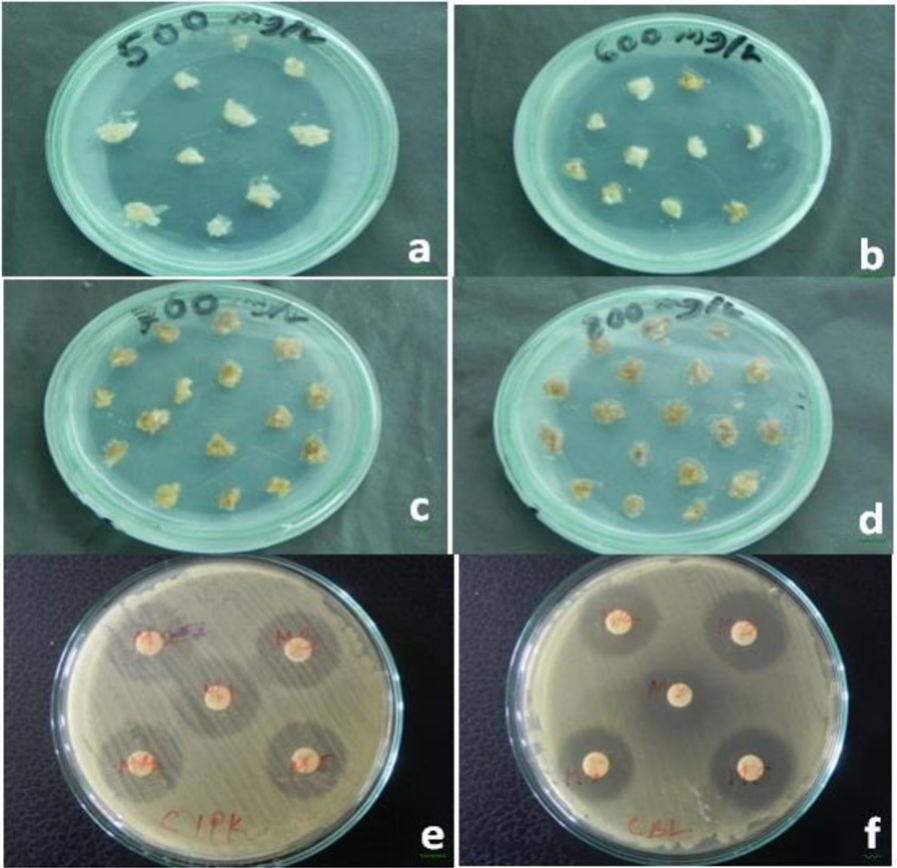
Antibiotic sensitivity test of explants and antibiogram of *Agrobacterium* strains. After seven days of inoculation of explants remained fresh (**a**) at 500 mg/l cefotaxime but became pale (**b**) at 600 mg/l cefotaxime and died at 700 mg/l, 800 mg/l (c, d). Clear zone were found at 500 mg/l cefotaxime in case of both strains *PsCIPK* (**e**) and *PsCBL* (**f**).

**Fig. 4:**
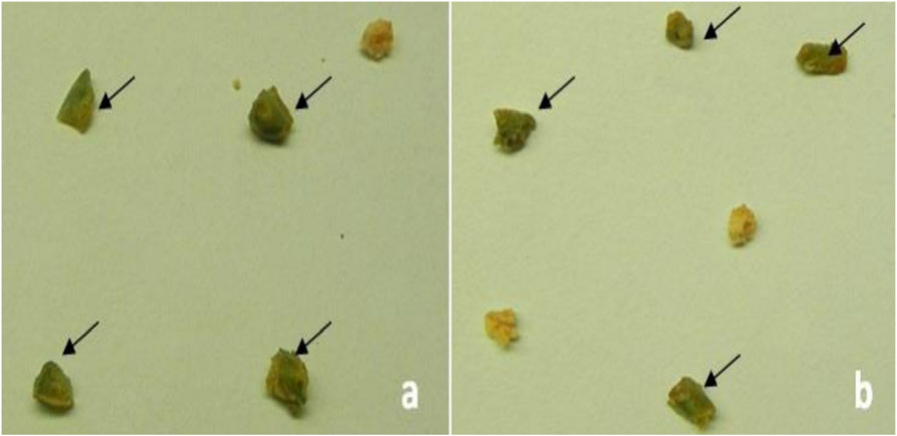
Assay of GUS activity in transformed calli of *Citrus reticulata Blanco* (**a**) and *Citrus sinensis* (L.) Osbeck (**b**). Arrow indicates the GUS-positive calli (blue spot)

**Fig. 5:**
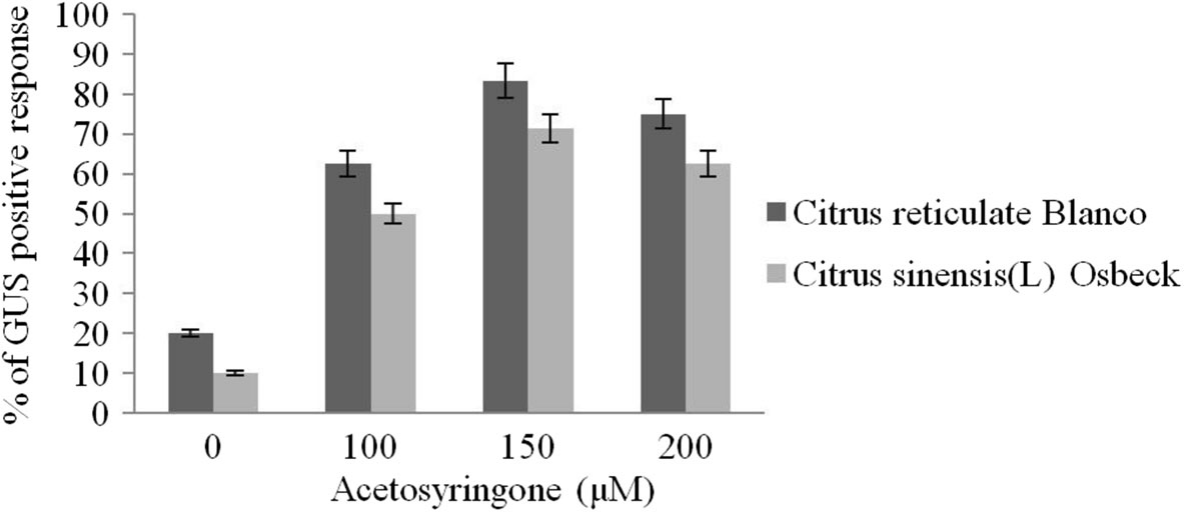
GUS assay of the putatively transformed calli in different concentrations of acetosyringone

**Fig. 6:**
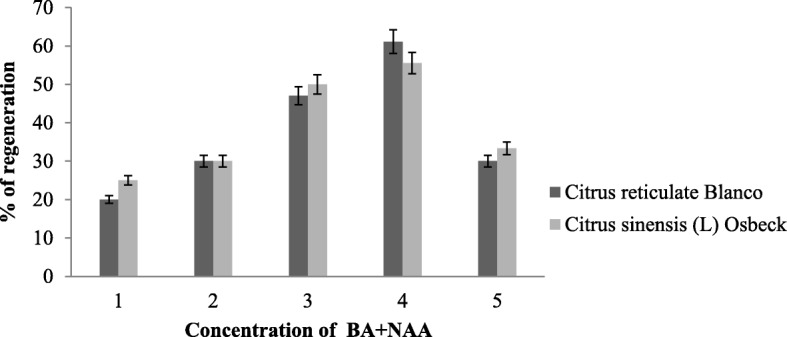
Effect of BA and NAA at different concentrations on regeneration of transformed calli Here, 1 = 2.0 BA + 1.0 NAA; 2 = 2.5 BA + 1.0 NAA; 3 = 3.0 BA + 1.5 NAA; 4 = 3.0 BA + 2.0 NAA; 5 = 3.5 BA + 2.5 NAA mg/l

**Fig. 7:**
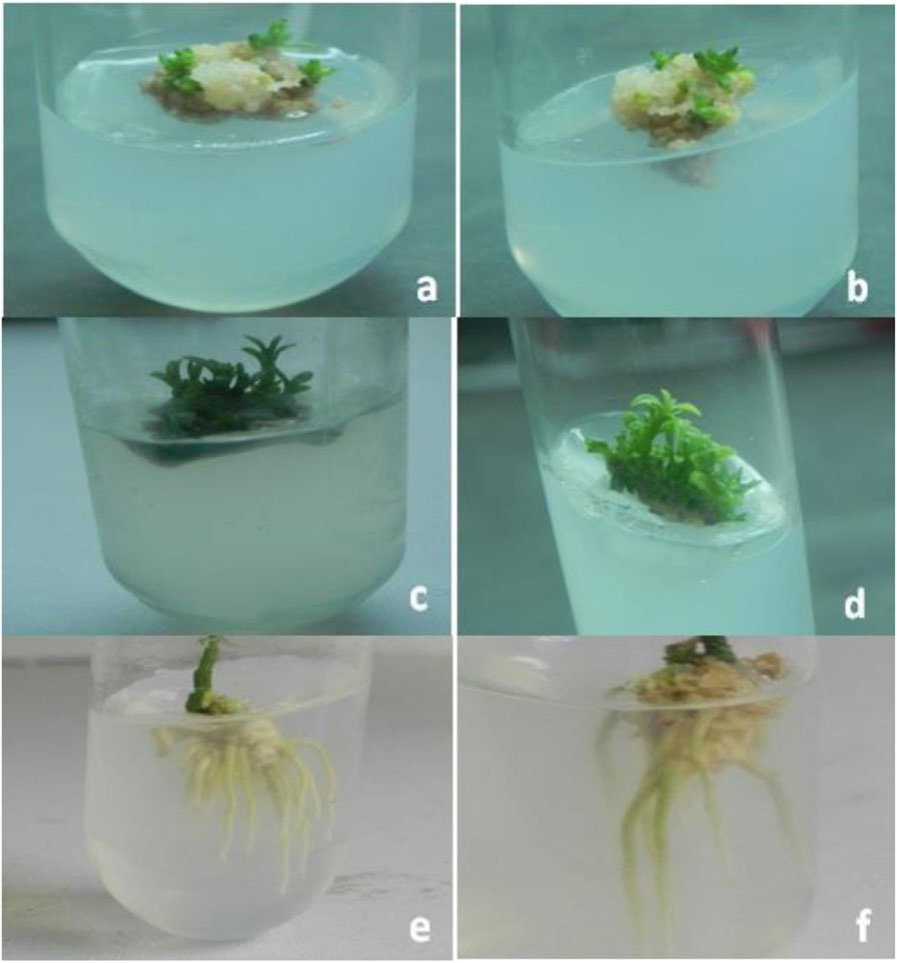
Shoot and root induction from kanamycin resistant explants on MS medium of *Citrus reticulata* Blanco (**a**, **c**, **e**) and *Citrus sinensis* (L.) Osbeck (**b**, **d**, **f**)

**Fig. 8:**
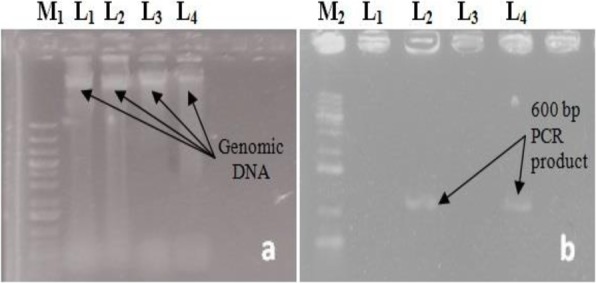
**a** Represents the genomic DNA and **b** represents the PCR product of the GUS gene. Lane M_1_: 1 kb plus ladder DNA and M_2:_ 1 kb ladder DNA; L_1_: DNA of control *Citrus reticulata* Blanco. L_2_: DNA of transformed *Citrus reticulata* (L.) Blanco. L_3_: DNA of control *Citrus sinensis* (L.) Osbeck. L_4_: DNA of transformed *Citrus sinensis* (L.) Osbeck

**Fig. 9:**
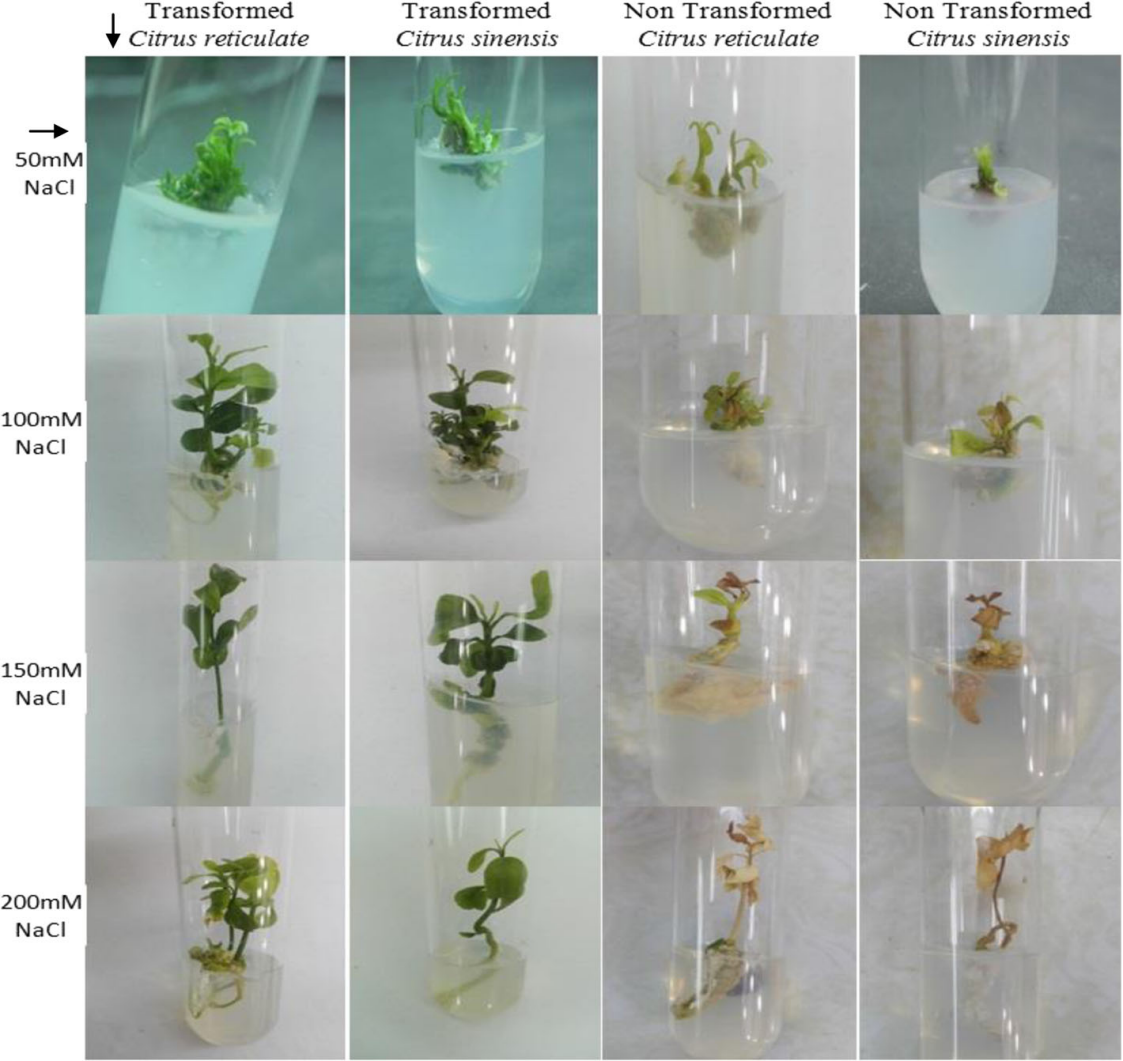
In vitro evaluation of transgenic and non-transgenic plantlets on MS medium containing different concentration of NaCl and 3 mg/l PEG after 10 days of inoculation

Mature dehusked seeds from *Citrus reticulata* Blanco and *Citrus sinensis* (L.) Osbeck were sterilized and inoculated on MS medium containing different concentrations of 2,4-D. After 3 weeks of incubation, yellowish white, light green, and green colored globular calli were generated (Fig. 1). Maximum callus induction frequency of *Citrus reticulata* Blanco and *Citrus sinensis* (L.) Osbeck 89.33 ± 3.03% and 95.67 ± 4.04%, respectively, was found when MS medium was supplemented with 3 mg/l 2,4-D (Fig. 2). Yellowish white colored globular calli were selected for transformation. The selected calli of both species were pre-cultured on the same media for 3 days. Then pre-cultured calli were infected by immersing them in the infection medium (MS) containing both *Agrobacterium* strains for 90 min with intermittent gentle shaking and dried on sterile filter paper for 30 minutes. After 3 days of co-culture in dark on co-culture medium, calli were washed with 500 mg/l cefotaxime and transferred onto selection medium for fifteen days and was sub-cultured again on same selection medium (Fig. 1). The concentration of antibiotic was optimized via antibiogram of *Agrobacterium* strains and plant tissue sensitivity test (Fig. 3). At 500mg/l cefotaxime, clear zones were found and no harmful effect on plant tissue (callus) was observed after 7 days of treatment. After the second sub-culture, the GUS-histochemical assay was done and the frequency of GUS-positive calli was 83.33 ± 2.42% and 43 ± 2.00% for both species respectively (Fig. 4). Maximum GUS-positive results were found when 150-μM acetosyringone was added to the co-culture media and incubated for 3 days (Fig. 5). The fresh kanamycin-resistant calli were then transferred to the regeneration medium and sub-cultured on same media at an interval of 21 days. After 4 weeks of inoculation on regeneration medium, shoot (2–10 shoot per callus) generation started (Fig. 6). The frequencies of shoot induction were 61.11 ± 1.35% and 55.5 ± 1.03% for both species respectively, when MS medium was supplemented with 3.0 mg/l of BA and 2 mg/l of NAA (Fig. 7). The shoots obtained from differentiation of callus were taken and cultured on rooting medium [17]. Roots appeared within 20 days of inoculation. The genomic DNA was isolated from leaves of the putative transformed plantlets and non-transformed control plants and run on a 0.8% agarose gel for each sample (Fig. 8a). Transformation at genomic level was detected by amplifying GUS gene. Specific primers were used in PCR analysis to verify the presence of transgenes (GUS). Transgenic plantlets of both species produced bands of the expected size of 600 bp (Fig. 8b), whereas the corresponding bands were not found in control plants. The presence of reporter gene at the genomic level confirms the transformation of abiotic stress tolerant genes in two *Citrus* species. This result confirms the stable transformation of the transgenes and successful integration in the genome. In-vitro evaluations of the transgenic plants were performed in MS medium supplemented with different concentrations of NaCl (50, 100, 150, and 200 mM). The transgenic plantlets of two species remained fresh and survived for more than 4 weeks on MS medium supplemented with 50 mM NaCl, 2 weeks on 100 and 150 mM NaCl, and 1 week on 200 mM NaCl but control plantlets became pale within 5–7 days and died after 10 days (Fig. 9). These results confirm the expression of stress tolerant genes in these *Citrus* species.

## Discussion

In this research, *Agrobacterium*-mediated *PsCBL* and *PsCIPK* genes were transferred to citrus species, *Citrus reticulata* Blanco, and *Citrus sinensis* (L.) Osbeck using mature seed-derived calli. The previous study [18] reported that mature seed-derived callus was the most amenable explants for *Agrobacterium*-mediated transformation. Differences between various hormone supplementations were observed in callus induction. The efficiency of the *Agrobacterium*-mediated transformation method was inspected through vigilant observations of the effects of several parameters considered to be critical. The success of transformation was assessed by the percentage of blue spots signifying transient expression of GUS gene and the PCR detection of GUS gene. The density of *Agrobacterium* directly affects transformation efficiency since gene transfer only occurs with proper *Agrobacterium* attachment to plant cells. Therefore, high *Agrobacterium* concentration increased the number of plant cells being infected [19]. In this study, the highest number of GUS staining was observed in *Agrobacterium* suspension OD_600_ at 0.5. More concentrated *Agrobacterium* suspension (when OD_600_ was 0.8–1.0); however, significantly reduced the number of transformed cells due to the fact that intense *Agrobacterium* infection caused severe damage to the plant cells. The previous study [19] reported that bacteria concentration at OD_600_ 0.6 to 0.8 was the most efficient in sweet potato embryogenic callus *Agrobacterium*-mediated transformation. Karami, 2008 [20] also reported that *Agrobacterium* concentration for transformation is dependent on multi-factors including *Agrobacterium* strain and viability, plant species, and cultivar and type of tissue used. The age of the callus is a crucial factor for transformation efficiency. Hiei and Komari, 2008 [21] reported that fresh and healthy immature embryos ensure the successful transformation. Young embryogenic callus is also favorable due to its higher regeneration ability as compared to old calli [22, 23]. Sharawat, 2007 defined the pre-culture period as the period that starts at the moment when immature embryos are first isolated and cultured immediately before *Agrobacterium* infection [24].

Zuraida et al. 2011 [25] showed that pre-culture period for more than 3 days improved transformation frequency. Four-day pre-cultured calli were used for *Agrobacterium* infection. During infection, the pre-culture calli were immersed in the infection medium for 90 mins with intermittent gentle shaking. The infected calli were dried for 30 mins on sterilized filter paper. Eighty-five transformation rate was recorded when calli were infected for 90 mins and dried for 30 mins [25]. In this study, infected calli were co-cultured for 3 days [25]. Zuraida et. al., 2011 stated a significant increase of transient GUS expressions were observed in 4 days of co-cultured calli but we found maximum GUS expression when co-cultured for 3 days. The influence of various acetosyringone concentrations (0, 100, 150, and 200 μM) in the co-culture medium were evaluated and significant GUS staining was achieved at the addition of 150 μM acetosyringone. A similar result was obtained in the transformation of sweet potato when the effect of acetosyringone concentration in co-cultivation medium was investigated [26]. After 3 days of co-cultured at the dark condition, the calli were washed with a washing solution containing 500 mg/l cefotaxime. The antibiotic concentration was selected through antibiogram of *Agrobacterium* and plant cell sensitivity test. Transient GUS gene expression was determined by GUS assay and PCR amplification of the GUS gene [27]. Almeida et al. 2003 stated that 81.5% GUS-positive result found in the *Agrobacterium*-mediated transformation of “Hamlin” sweet orange which is nearly similar to our result. Media composition, mainly the hormonal balance is an important factor influencing in-vitro culture initiation and plant regeneration from callus [28]. MS medium supplemented with 3 mg/l of 6-benzylaminopurine (BA) showed maximum regeneration efficiency for both *Citrus* species [29]. reported that the MS medium supplemented with 3 mg L-1 of 6-benzylaminopurine (BA) showed maximum regeneration efficiency of the transformed explants. Shoots were rooted on MS medium without supplementation of hormone [29–32] and the rooted plantlets were evaluated on stress conditions. The transgenic plantlets showed moderate resistance against abiotic stresses like salt.

## Conclusion

The demand of *Citrus* (orange) is increasing day by day in Bangladesh. A huge amount of foreign currency is being spent for importing orange. It is possible to grow *Citrus* commercially, fulfill the national demand, and save foreign currency by eliminating the problems by developing good varieties. In this research, a reliable and efficient transformation system for two *Citrus* species was developed and transgenic plants with stable integration of target genes were recovered. This study will help for the development of *Citrus* varieties with desired traits.

## Data Availability

Authors declare that all generated and analyzed data are included in the article.

## Abbreviations

2,4-D: 2,4-Dichlorophenoxyacetic acid
BA: 6-Benzylaminopurine
GUS: *β-*glucuronidase
MS: Murashige and Skoog
NAA: 1-Naphthaleneacetic acid
*nptII*: Neomycin phosphotransferase II
OD_600_: Optical density at 600 nanometer UV light
PCR: Polymerase chain reaction
*PsCBL*: Calcineurin B-like protein of *Pisum sativum*
*PsCIPK*: CBL-interacting protein kinases of *Pisum sativum*
UV: Ultraviolet
